# How can a behavioral economics lens contribute to implementation science?

**DOI:** 10.1186/s13012-024-01362-y

**Published:** 2024-04-26

**Authors:** Nathan Hodson, Byron J. Powell, Per Nilsen, Rinad S. Beidas

**Affiliations:** 1 Price School of Public Policy, University of Southern California, Los Angeles, USA; 2 Warwick Medical School, Unit of Mental Health and Wellbeing, Division of Health Sciences, University of Warwick, Coventry, UK; 3 Brown School, Center for Mental Health Services Research, Washington University in St. Louis, St. Louis, USA; 4 Center for Dissemination & Implementation, Institute for Public Health, Washington University in St. Louis, St. Louis, USA; 5 Division of Infectious Diseases, John T. Milliken Department of Medicine, School of Medicine, Washington University in St. Louis, St. Louis, USA; 6 Department of Health, Medicine, and Caring Sciences (HMV), Linköping University, Linköping, Sweden; 7 School of Health and Welfare, Halmstad University, Halmstad, Sweden; 8 Department of Medical Social Sciences, Feinberg School of Medicine Northwestern University, Chicago, USA; 9 Center for Dissemination and Implementation Science, Institute for Public Health and Medicine, Chicago, USA

**Keywords:** Behavior change, Behavioral economics, Interdisciplinary research, Implementation strategies, Implementation frameworks

## Abstract

**Background:**

Implementation science in health is an interdisciplinary field with an emphasis on supporting behavior change required when clinicians and other actors implement evidence-based practices within organizational constraints. Behavioral economics has emerged in parallel and works towards developing realistic models of how humans behave and categorizes a wide range of features of choices that can influence behavior. We argue that implementation science can be enhanced by the incorporation of approaches from behavioral economics.

Main body

First, we provide a general overview of implementation science and ways in which implementation science has been limited to date. Second, we review principles of behavioral economics and describe how concepts from BE have been successfully applied to healthcare including nudges deployed in the electronic health record. For example, de-implementation of low-value prescribing has been supported by changing the default in the electronic health record. We then describe what a behavioral economics lens offers to existing implementation science theories, models and frameworks, including rich and realistic models of human behavior, additional research methods such as pre-mortems and behavioral design, and low-cost and scalable implementation strategies. We argue that insights from behavioral economics can guide the design of implementation strategies and the interpretation of implementation studies. Key objections to incorporating behavioral economics are addressed, including concerns about sustainment and at what level the strategies work.

**Conclusion:**

Scholars should consider augmenting implementation science theories, models, and frameworks with relevant insights from behavioral economics. By drawing on these additional insights, implementation scientists have the potential to boost efforts to expand the provision and availability of high quality care.

Contributions to the literature

- Implementation scientists regularly draw on insights from diverse fields of study but have not systematically incorporated findings from behavioral economics.
- We illustrate how a behavioral economics lens can enhance traditional implementation strategies and inform interpretation of implementation studies, going further than classical behaviour change theories.
- Insights from behavioral economics have the potential to “supercharge” or “boost” implementation science, thus we call for closer integration between the two literatures.

## Introduction

Human behavior is the last mile challenge to many seemingly intractable problems in improving the human condition. Many scientific discoveries are unevenly accessed and delivered due to an underappreciation for how social and behavioral factors might interface with the implementation of these discoveries. For example, during the COVID-19 pandemic, mRNA vaccines were developed and available within one year and yet too little consideration was paid to implementation and human behavior, resulting in uneven implementation and stark inequities [1]. Similarly, as it becomes clear that some prescribing practices are low-value, the de-implementation process requires changing clinician behavior [2, 3]. As new screening modalities emerge that can prevent the onset of disease, it is essential that clinicians refer those patients most likely to benefit [4].

Implementation science has emerged as a convergence field, bringing together multiple disciplines to close the gap between what we know and what we do – or in other words, focused on behavior change of healthcare actors within organizational constraints, including clinicians, managers, funders, and health service users [5]. Implementation science has made great advances in coalescing as a field over the past two decades, drawing on a range of disciplines including organizational theory, human factors, improvement science, and adult learning theory [6]. Early work in implementation science characterized barriers and facilitators to implementation when initial efforts to change behavior within organizational constraints were often unsuccessful. The next generation of studies explored cross-sectional relationships between determinants hypothesized in leading conceptual frameworks. The most recent work in the field tests the comparative effectiveness of implementation strategies [7]. However, limitations of the current paradigm include an overreliance on education-focused implementation strategies such as training [8], approaches that are designed for the ideals of human behavior and do not take into account knowable and predictable patterns of human decision making, and the use of costly resource-intensive strategies that are difficult to scale.

The field of behavioral economics has developed in parallel and has also focused empirical inquiry on understanding human behaviors within various settings, including the healthcare environment. Behavioral economics offers a paradigm shift in how social scientists, including psychologists and economists, understand human behavior and decision-making [9]. Through 50 years of research, novel findings in psychology and economics have allowed behavioral economists to identify and categorize factors that drive human behavior in surprising but replicable ways, disrupting existing scholarly consensus about how people make decisions and introducing a new set of frameworks for researchers and policymakers. Importantly, behavioral economics offers simple and low-cost approaches that build on evidence of how humans make decisions. Specific concepts from behavioral economics that have been applied to change health behavior include promoting vaccination [10], de-prescribing [11], and screening [12]. For example, informing members of the public that a personal COVID-19 vaccine was ready specifically for them was effective at increasing vaccination uptake [10]. Default settings in electronic health records have changed prescribing behavior [2]. And recent scholarship has highlighted the scope for improving cancer screening pathways by removing unnecessary friction and paperwork, sometimes described as ‘sludge’ [4, 13].

While focused on similar outcomes, the two fields have not been explicitly woven together, thus offering an opportunity for synergizing and maximizing impact. Systemically incorporating the behavioral economics perspective into implementation science is an important opportunity to advance the field. We propose that approaches from behavioral economics can allow us to understand behaviors in a more complete and nuanced manner (See note 1). More specifically, we argue that insights from behavioral economics can guide the design of implementation strategies and the interpretation of implementation studies for the advancement of the field.

### Behavioral economics explains behavior in a more realistic and practical way

Classical economic theories of human behavior assume humans maximize utility – in other words appraising all potential actions and selecting the one perceived to be the most beneficial – but evidence from behavioral economics has increasingly revealed that humans do not maximize utility when making decisions [9, 14]. That is to say, given human decision-making processes, all people working in healthcare are “boundedly rational” in predictable ways [15]. Dual Process Theory is one important way of understanding this phenomenon, holding that people make decisions using two systems. System 1 is fast, intuitive, and automatic, and prioritizes efficiency. It relies on heuristics, or mental shortcuts, and biases which are commonly repeated patterns of responses [9, 16]. System 1 runs without us noticing but is prone to errors because its heuristics and biases are generally useful but not tailored to every situation. System 2 is slow, conscious, and effortful. It analyses problems logically to avoid pitfalls, but tires quickly [9].

The predictable thinking patterns or biases which result in bounded rationality are increasingly well-described and replicated. They impact almost every part of life, including many areas of healthcare. *Present bias*, for example, means that people prefer immediate pleasure compared with delayed pleasure; we tend to accept more pain later rather than a little immediate pain [17]. This might mean patients avoid vaccinations now despite risking hospitalization later. It could mean clinicians delay learning about a technology which will improve the efficiency of their practice because they anticipate that the initial process of learning will be tiring and stressful. Due to *commission bias*, people tend to choose to act rather than not act [18]. This could lead to doctors recommending cancer screening for patients at low risk of cancer because it feels like they are taking action whereas creating cancer scares, unnecessary biopsies, and private pain or loss of function is harmful and low-value care. Under the *availability heuristic* people consider outcomes more likely if they can readily bring them to mind; for example, people can easily imagine an airplane crash but less readily imagine chronic lung disease, and so they exaggerate their likelihood of dying in the former and underrate the risk of the latter [19]. This misperception might lead to reduced engagement with preventive efforts like smoking cessation support [20]. The same effect might make doctors excessively risk-averse by endowing rare adverse outcomes with an outsized impact on decision-making.

The extent to which heuristics and biases impact behaviors is mediated by the environment. Behavioral economists use the term *choice architecture* for the whole range of features of the environment that shape behaviors [21] including default options, positive or negative framing, reminders, social factors (who is watching and what others are doing), cognitive factors (other concurrent decisions and ease of access to data), and uncertainty (regarding information or regarding outcomes) [14, 22, 23]. While a decision maker who is maximizing utility would optimally pursue their preferences irrespective of the choice architecture, human behavior is frequently influenced in predictable ways when choice architecture interacts with heuristics and biases.

This rich understanding of how humans think, feel, behave, and make decisions can allow choice architects to help people make better decisions. One way of doing this is through “nudging” or altering the choice architecture to help people make better decisions (although the implications of behavioral economics go much further) [21, 24, 25]. Autoenrollment into pension savings is one example of a nudge [26]. Over the last decade the number of people in the UK saving for retirement has increased significantly because, rather than being asked to opt-in to pensions, they were assumed to want to save, automatically signed into the program, and given the chance to opt-out [27]. This changed outcomes because people making decisions with system 1 tend to stick with the default: at first the default was non-enrollment, now the default is enrollment. In some jurisdictions organ donation has undergone a similar change. [28]. Choice architecture exists whether we intentionally design it or not. For example, if non-enrollment in organ donation is the default, it is still a default. It is incumbent on people designing choice architectures to consider whether predictable patterns of human behavior due to heuristics and biases will interact with features of the choice architecture in ways that help or hurt people.

Another way of leveraging insights from behavioral economics to help people make better decisions is to remove features of choices which are cognitively or emotionally draining, sometimes called “sludge” [24]. The term “cognitive misers” is sometimes used to describe how humans have a universal tendency to make decisions that conserve cognitive and emotional energy, and thus sludge can stop people making choices they would otherwise want to make [29, 30]. Examples of sludge include when a second-hand car dealer makes a customer sign a disclaimer before allowing them to decline an overpriced insurance add-on or when it is difficult to access naloxone in the event of opioid overdose. Since 2016, the state of California has made it legal for pharmacists to dispense naloxone without a prescription [31]. Desludging healthcare by making systems quick and straightforward to use can help clinicians, healthcare administrators, and patients make better decisions.

The findings of behavioral economics suggest that policy should draw on evidence of how humans actually behave. In his book *Inside The Nudge Unit*, David Halpern emphasized the importance of a more realistic model of behavior [32].

*"A practical approach to government, or business, based on a realistic model of people would be messier than that of traditional economics or law. It would need to reflect the complexity of the human mind – what we do well, and what we don’t. It would imply thinking of cognitive capacities as wonderful, but precious resources. When we design services and products, we would need to be respectful of this reality, and remember that people have generally got better things to do than wade through bureaucracy or the puzzling ‘rationality’ of the state or big business. We would have to design everything we do around people, not expect people to have to redesign their lives around us.” *[32]

Halpern put this approach into practice at the Behavioural Insights Team in the UK Civil Service, but this description also indicates how implementation science could be informed by behavioral economics. Applying a behavioral economics lens entails drawing on empirical evidence to ensure implementation science is informed by an awareness of what the human mind does well, where it struggles and tires, and how people respond to different choice architectures. It means considering how humans really behave within the context of implementation, not how we hope they will behave.

### General insights from behavioral economics on decision-making have been largely overlooked in implementation science

Implementation scientists have largely overlooked the impact of bounded rationality on decision-making [8, 33]. Clinicians are expected to change behavior as new practices or policies are introduced within their organizational contexts and patients are expected to adhere to relevant advice or medication. In other words, the fundamental assumption is that knowledge is a major mechanism of behavior change. However scholarship demonstrates that knowledge may be necessary but is rarely sufficient for behavior change [8]. Therefore, most implementation endeavors stand to benefit from considering a behavioral economics lens, which could include defaults, cognitive bandwidth, motivated reasoning, or any of the other areas where research in behavioral economics is relevant for understanding the challenges of implementing new practices that require behavior change. For example, altering electronic prescribing systems such that the preferred prescribing option is selected automatically is using a *default* to increase evidence-based prescribing [34].

Several well-known implementation science models draw on social-cognitive theory, such as Theoretical Domains Framework (TDF), Capability Opportunity Motivation Behaviour (COM-B) and Clinical Performance Feedback Intervention Theory (CP-FIT). These theories incorporate rich conceptions of human behavior and decision-making, but do not explicitly move beyond the assumption that we are mindful and deliberate in all our actions [35, 36]. The full depth of relevant findings about human behavior, particularly how heuristics and biases influence behavior in surprising but replicable ways, has not yet been systematically integrated with implementation science. This gap presents an opportunity to design strategies that account for how humans actually behave rather than how we hope they will behave, and to enrich our understanding of the challenges of changing behavior and influencing decision-making as new practices are implemented [35–37]. Emerging work in this space has not yet been fully embedded within the general implementation science approach but represents a promising direction for forward movement [38, 39]. Table 1 outlines how the behavioral economics lens can inform implementation science.

**Table 1:** What does a Behavioral Economics Lens offer to IS?

	**IS**	**What does a Behavioral Economics Lens offer to IS?**
Focus and key assumptions	Multi-level approaches for Improving adoption and integration of evidence-based practices into real world settings [5] with an emphasis on individual behavior change and decision making within organizational constraints	Designing for human behavior and clinician decision-making based on the principle that humans have bounded rationality
Research methods	Qualitative and quantitative research methods to improve implementation	Offering additional methods including pre-mortems (prospectively leveraging the power of hindsight bias)[45] and behavioral design (explicitly mapping out conscious and unconscious behavioral barriers and designing for those barriers)
Approach to context	Focus on multi-level contexts[60–62]	Particular emphasis on how choice architecture impacts individual-level decision-making [55]
Starting point	Generally begins with comprehensive and intensive implementation strategies as standard	Starts with low cost and scalable solutions and layering on more resource-intensive approaches as required
Strategies to behaviour change	Increasingly matching implementation strategies to barriers identified by participants [63]	Designing choice architecture that makes evidence-based practices the default or makes them salient, creates competition, or makes the evidence-based practice easier, attractive, social, or timely – and by accounting for barriers that might arise due to heuristics and biases

#### The behavioral economics lens can guide the design of implementation strategies

To move towards integrating behavioral economics approaches in implementation science, we must evaluate the role bounded rationality and behavioral factors play in implementation to optimally design implementation strategies. By capitalizing on evidence of the impact of heuristics and biases and the potential impact of choice architecture, the behavioral economics lens can help with better design of implementation strategies to change behavior at multiple levels, including the individual, team, profession, and organization. Over recent years implementation scientists have adopted systematic approaches to selecting implementation strategies, drawing on logic models and taxonomies of implementation strategies, along with implementation mapping [40]. These approaches have strengthened the field by targeting specific contextual barriers identified by constituents. Behavioral design takes this a step further by incorporating the biases and heuristics which can influence behavior but may remain outside of constituents’ conscious awareness [41]. Two behavioral economic typologies, EAST and MINDSPACE offer guides for researchers considering incorporating heuristics and biases into implementation strategy design [14, 42].

EAST incorporates four domains where behavioral insights apply: easiness, attractiveness, social factors, and timing. Considering these domains can guide scholars designing implementation approaches to consider whether their approach capitalizes on recognized ways of changing human behavior. These four elements can be used in different ways, for example, men’s health interventions like Movember have drawn on social factors by prompting conversations about men’s health, whereas the Do It For Babydog vaccination campaign in West Virginia gave children chance to win a party for their whole school, creating a different kind of social impetus [43, 44]. Conversely, considering the four EAST domains at the design stage can bring to attention overlooked features. This can be seen when implementation strategies include incentives: deferred incentives are less powerful than immediate incentives, so consideration of the timing domain could help an implementation designer consider ways of offering a reward alongside the behavior rather than waiting until later.

Whereas EAST provides factors to consider, MINDSPACE gives a specific list of strategies. This more extensive typology lists simple nudges, such as incentives, norms, and defaults, that implementation scientists may consider when looking for ways to boost behavior change. In applying a behavioral economics lens, the implementation scientist might review the MINDSPACE framework to find an appropriate technique. The implementation designer may decide that it would be appropriate to include “Commitments” by inviting participants to make a public promise to change practice. Alternatively, the pre-mortem approach can be used to leverage the power of prospective hindsight, where team members imagine an implementation effort has already failed and discuss all the causes of the failure, to make potential mitigation targets more salient[45, 46]. In each case, EAST helpfully draws attention to domains where the behavioral economics lens might apply and the MINDSPACE framework offers specific strategies which could be incorporated.

One study that illustrates how to apply these concepts to the design of implementation strategies was conducted by Patel and colleagues [2]. A change in the choice architecture where the default became generic medications vs. the previous default (i.e. name-brand medications) resulted in a 5 percentage-point greater increase in default prescriptions. Large changes were noted in prescriptions where there was little clinical difference between preparations but smaller changes in prescriptions such as thyroxine and, to a lesser extent, anti-epileptics, demonstrating that prescribers overrode defaults and maintained agency where there was a clinical indication [47]. In this case, Patel et al*.* focused on easiness from the EAST framework, and used the default approach from the MINDSPACE repertoire.

### The behavioral economics lens can inform the interpretation of implementation trials

Post-implementation evaluation can also be informed by the behavioral economics lens. Making sense of the outcomes of studies of different implementation strategies is not straightforward, particularly when unexpected results arise [48]. Evaluation studies often incorporate mixed qualitative and quantitative methodologies which benefit from a theoretical framework, and the behavioral economics lens provides one such framework. Post-trial evaluations perform the crucial function of appraising the replicability of findings and may note extraneous factors which influenced the study, such as changes to the organizational or national context. Similarly, surprising findings may be explained by behavioral factors. For example, an otherwise effective implementation may be undermined by a default policy or norm; identifying such behavioral factors allows implementation scientists to offer more insightful advice for future studies.

Purtle et al*.*’s recent dissemination study illustrates this approach [49]. The authors explored whether state legislators were more likely to open emails which contained local economic data about the impact of adverse childhood experiences (ACEs). Purtle et al*.* found in secondary analysis that Democratic legislators were more likely to open emails labelled as containing useful economic data about ACEs, whereas Republican legislators were no more likely to open those emails than emails offering no economic data. The authors briefly discussed motivated reasoning, a concept incorporated into behavioral economics from the social cognition literature, describing how a desire to hold certain beliefs influences the way people seek out and evaluate sources of information [49, 50] (see note 2). Information avoidance, a concept closely related to motivated reasoning, helps make sense of their surprising findings: people tend to avoid finding out facts that threaten their existing pre-existing beliefs [51]. If Republicans are in general wary of government intervention in family life, they may avoid information which implies government should act around ACEs. Purtle et al*.*’s use of the behavioral economics lens is innovative and provides a model for others trying to make sense of unexpected findings.

Another recent study by Glidewell et al*.* evaluated the success of several strategies for changing practice in UK primary care. Their interpretation of the results was implicitly in keeping with common findings from behavioral economics [52]. The authors found strong evidence of information avoidance in their less successful implementations; where searches of patient lists were expected to create an unmanageable amount of work, administrative staff admitted not conducting the searches. Glidewell et al*.* appear to have drawn on insights from behavioral economics in their design and interpretation of this paper illustrating the usefulness of the behavioral economics lens. However, they did not cite the behavioral economics literature or use terminology from behavioral economics. While these omissions are reasonable when communicating only with other implementation scientists, they offer a missed opportunity to use shared keywords that would allow behavioral economists to benefit from frontline applications of these theories. Behavioral economics brings together ideas such as social comparison and information avoidance from other disciplines such as social psychology; using the same terminology can also enrich implementation science and provide useful explanations for better understanding findings that benefit from a behavioral lens.

### Objections to behavioral economics

While behavioral economics offers new insights to boost implementation science, it is important to highlight potential limitations to this approach.

While some implementation efforts such as those around prescribing change are located close to the individual choices of clinicians and are highly amenable to a wide range of behavioral interventions, many implementation efforts relate to team or organizational behavior. Behavioral economics is also relevant within the multilevel nature of implementation science, as many of the core ideas in behavioral economics relate to the way groups and teams work and how colleagues relate to each other. Common approaches to categorizing behavioral biases highlight the importance of attempts to change behavior by making desired actions social and taking into consideration how group norms can be presented [14]. Behavioral economists have also explored how bounded rationality alters group decision-making, team coordination and colleague effort levels. Across teamworking tasks, coordination tasks, and competitive tasks, behavioral economists have found surprising results which could not be explained by traditional economic models [53]. For example, when colleagues were randomly paired for an effort task they tended to perform equally, specifically because the lower performing participant worked harder to match the higher performer. However, more work is needed to apply these concepts to the team and organizational levels.

Similarly, the importance of sustainment is increasingly recognized by implementation science [54]. Interventions from behavioral economics vary by duration of behavior change. For example, removing sludge or setting default options can influence behaviors repeatedly [14]. Other behavioral insights such as social comparison nudges may only influence behavior while the intervention is actively being managed, just as an implementation strategy with a didactic education component is difficult to sustain as new staff arrive unless sustainment has been actively managed. Implementation scientists drawing on the behavioral economics lens should consider which elements match the duration of behavior change sought.

Finally, some behavioral economists have recently warned that prioritizing nudging over all other interventions could distract policymakers’ attention from the underlying drivers of adverse policy outcomes such as structural inequities [55]. Scholars have noted the importance of using integrated strategies which combine nudges with political and social approaches to improve outcomes broadly and equitably [56]. In public health contexts such an approach could entail combining individual behavior change strategies with advocacy for investment in equitable access. It is important that implementation scientists remain alert to outer context factors when drawing on the behavioral economics lens and we anticipate that the field is well positioned to avoid this potential limitation of behavioral economics [57].

## Conclusion

Implementation science has rapidly attained influence and respect because of its positioning as a convergence field bringing together transdisciplinary approaches to closing the gap between what we know and what we do. Notably, the behavioral economics lens has featured little within the development of the field. We have argued that implementation science can now be enhanced by the incorporation of approaches from behavioral economics, particularly by considering heuristics and behavioral biases that shape decision-making and behavior and by leveraging these known, predictable patterns to design choice architecture within the context of implementation strategy design.

This opportunity to integrate a behavioral economics lens into implementation science merits continued attention and consideration. If otherwise well-designed implementation strategies are undermined by behavioral economic phenomena there is a risk that the field of implementation science will have reduced impact. Just as the AACTT framework (action, actor, context, target, and time) has helped implementation scientists report intended behavior change mechanisms with greater clarity we suggest that explicit description of the changes to choice architecture and their anticipated effect on behavior would help other implementation scientists to evaluate and replicate implementation approaches [58]. To make this easier for other implementation scientists, scholars may consider augmenting implementation science frameworks and taxonomies with relevant behavioral insights. With these additional frameworks, implementation scientists have the potential to supercharge efforts to expand the provision and availability of evidence-based practices. [59]

## Data Availability

Not applicable.

## Footnotes

1 Implementation science often focuses on clinician behavior change but we recognize implementation science is increasingly salient to how behavior change among a broad range of individuals and groups, including healthcare leaders, policymakers, and even patients, influences the extent to which evidence-based practices and policies are put into practice. For simplicity, we will generally refer to clinician behavior change in this article, but the principles and recommendations can be relevant to other actors’ behaviors [59].

2 It is important to note that Social Cognition is a blend of social and cognitive psychology and is different from Social-Cognitive approaches discussed above.
